# Simultaneous Rapid Detection of Aflatoxin B_1_ and Ochratoxin A in Spices Using Lateral Flow Immuno-Chromatographic Assay

**DOI:** 10.3390/foods10112738

**Published:** 2021-11-09

**Authors:** Xue Zhao, Xindi Jin, Zhang Lin, Qi Guo, Bin Liu, Yahong Yuan, Tianli Yue, Xubo Zhao

**Affiliations:** 1 College of Food Science and Engineering, Northwest A&F University, No. 22 Xinong Road, Yangling 712100, China; 15735172570@163.com (X.Z.); xd.jin@nwafu.edu.cn (X.J.); linzhang@nwafu.edu.cn (Z.L.); guoqiqi@nwafu.edu.cn (Q.G.); liubin7723@163.com (B.L.); yyh324@tom.com (Y.Y.); yuetl@nwafu.edu.cn (T.Y.); 2 Laboratory of Quality & Safety Risk Assessment for Agro-Products (Yangling), Ministry of Agriculture and Rural Affairs, Yangling 712100, China

**Keywords:** aflatoxin B_1_, ochratotoxin A, gold immuno-chromatography assay, spice, detection method

## Abstract

Spices are susceptible to contamination by aflatoxin B_1_ (AFB_1_) and ochratoxin A (OTA), which are both mycotoxins with high toxicity and carcinogenicity. In this study, we aimed to develop an immuno-chromatographic strip test for the simultaneous quantification of AFB_1_ and OTA in spices by spraying the coupled antigens AFB_1_–ovalbumin (AFB_1_–OVA) and OTA–ovalbumin (OTA–OVA) on a nitrocellulose membrane. The test strip had high sensitivity, good specificity, and strong stability. The detection limits of these two mycotoxins in Chinese prickly ash, pepper, chili, cinnamon, and aniseed were 5 μg/kg. The false positivity rate was 2%, and the false negativity rate was 0%. The maximum coefficient of variation was 4.28% between batches and 5.72% within batches. The average recovery rates of AFB_1_ and OTA in spices were 81.2–113.7% and 82.2–118.6%, respectively, and the relative standard deviation (RSD) was <10%. The actual sample detection was consistent with high performance liquid chromatography analysis results. Therefore, the immuno-chromatographic test strips developed in this study can be used for the on-site simultaneous detection of AFB_1_ and OTA in spices. This method would allow the relevant regulatory agencies to strengthen supervision in an effort to reduce the possible human health hazards of such contaminated spices.

## 1. Introduction

Spices are usually produced in countries with tropical climates that have high temperatures, humidity, and rainfall [1], which are also favorable conditions for the growth of microorganisms. As traditional agricultural practices are followed in developing countries, the storage and processing conditions of spices are generally overlooked. Therefore, spices are considered important carriers of fungal contamination [2], which occurs at various stages of planting, harvesting, and storage. Under suitable conditions, certain fungi can produce mycotoxins, which are highly stable and heat-resistant metabolites unaffected by conventional cooking methods [3]. This poses a huge challenge to human food safety issues. Some studies have reported that the presence of two or more mycotoxins in food exerts a synergistic effect on food toxicity [4]. Although several types of mycotoxins are found in food, the main contaminants of spices are aflatoxins (AFs) and ochratoxin A (OTA) [5,6]. Aflatoxin B_1_ (AFB_1_) is the most toxic and carcinogenic substance among aflatoxins. Studies have confirmed that both AFB_1_ and OTA induce hepatotoxicity, immuno-toxicity, neurotoxicity, and teratogenicity [7]. The International Agency for Research on Cancer has classified AFB_1_ and OTA as human carcinogens belonging to groups 1 and 2B, which indicates that the available evidence on the carcinogenicity of OTA to humans is not sufficient for AFB_1_ [8]; however, this does not imply that OTA is less toxic than AFB_1_ [9]. Based on these hazards, the European Union stipulates that the maximum allowable limits of AFB_1_ and total AFs in spices are 5 and 10 µg/kg, respectively [10]; the maximum allowable limit of OTA is 15 µg/kg [11]. In the United States of America, the total limit of Afs is 20 µg/ kg [12]. Although these regulatory measures and limitations can control the contaminated spices from entering consumers’ homes, the fungal contamination of agriculture and crops during pre- and post-harvest conditions is inevitable. Thus, adopting a fast, accurate, and easy mycotoxin detection method is important for the prevention and control of mycotoxin contamination in spices.

To date, the detection methods of mycotoxins in spices mainly include an enzyme-linked immunosorbent assay (ELISA), high performance liquid chromatography (HPLC), and HPLC–mass spectrometry (HPLC–MS) [13,14,15]. Besides having high sensitivity and specificity, ELISA has low purity requirements for samples [16]; the separation efficiency of HPLC is high [17], and HPLC–MS samples require a simple pretreatment. These advantages make these three detection methods popular for food mycotoxin detection. However, to achieve low-cost, rapid, and low-demand on-site detection, it is necessary to rely on colloidal gold immuno-chromatography assay (GICA) [18], which is an emerging immunoassay that can directly perform a visual qualitative or semi-quantitative analysis of the target. The GICA test results are reflected by the color development of the lightweight lateral flow immuno-chromatographic analysis strip (ICA). Farmers without special training can also take them into the field and directly detect and classify crops before picking. Thus, GICA has potential for the detection of toxic and hazardous substances in food [19]. Since the use of multiple types of ICA alone increases the cost and time for detection [20], the research direction after 2012 has shifted toward developing a new type of ICA that can detect two or more target substances simultaneously. However, so far, only corn, wheat, and other light-colored, single-matrix samples that have almost no background interference caused by pigmentation have been widely studied [21,22]. Spices have a complex matrix because of their deep pigment, essential oils, and aromatic substances. This complex matrix and the associated cross-reactions could make the mycotoxin test results inaccurate and potentially even produce false-positive results. Therefore, the establishment of a rapid detection method based on GICA for the simultaneous detection of AFB_1_ and OTA in the complex matrix of spices has strong operability and practicality.

In this study, a rapid detection method for the simultaneous detection of AFB_1_ and OTA in spices was successfully established. In addition, the detection results of two mycotoxins in spices were compared by GICA and HPLC methods to ensure the reliability of the GICA method. This is the first time GICA has been used to simultaneously detect AFB_1_ and OTA in spices.

## 2. Materials and Methods

### 2.1. Reagents and Solutions

Methanol and acetonitrile (HPLC grade) were purchased from Merck Drugs and Biotechnology (New York, NY, USA). AFB_1_, AFB_2_, AFG_1_, AFG_2_, OTA, zearalenone (ZEN), trichothecenes (T_2_), deoxynivalenol (DON), fumonisins (FB) standard solutions, goat anti-mouse IgG, bovine serum albumin (BSA), and ovalbumin (OVA) were purchased from Sigma-Aldrich (St. Louis, MO, USA). Sodium borate decahydrate, potassium carbonate, sucrose, Tween-20, acetic acid, sodium azide, sodium chloride, sodium hydrogen phosphate, potassium dihydrogen phosphate, potassium chloride, hydrochloric acid, and sodium bicarbonate were purchased from Damao Chemical Reagent Factory (Tianjin, China). Colloidal gold (particle size ~60 mm) and AFB_1_/OTA monoclonal antibody were purchased from Clover Technology Group Inc. (Beijing, China).

The following reagents were used: PBS; boric acid buffer solution (0.2 mol/L); resuspension solution, gold label pad treatment solution, sample pad treatment solution, sample buffer [23].

### 2.2. Sample Collection

In total, 150 samples of Chinese prickly ash, pepper, chili, cinnamon, and aniseed (each sample exceeding 0.5 kg) were randomly purchased from supermarkets, urban–rural junctions, and free markets in a ratio of 1:2:3. The spices collected from supermarkets were all sealed and packaged samples. The spices collected from the urban–rural junctions and free markets were all bulk samples placed in the open. Samples were crushed and stored at 4 °C until further analysis.

### 2.3. Gold-Labeled Antibody Preparation

Colloidal gold solution (40 mL) was placed in a centrifuge tube; 80 μL of 0.2 mol/L potassium carbonate solution was added under continuous stirring. Then, 500 μg each of AFB_1_ monoclonal antibody and OTA monoclonal antibody were added, dropwise. The mixture was allowed to stand at 25–30 °C for 1 h. Subsequently, 4 mL of 10% BSA was added while stirring and allowed to stand for 20 min. This mixture was centrifuged at 16,260× *g* for 30 min at 4 °C, and the supernatant was discarded. Forty microliters of a boric acid buffer solution containing 1% BSA at a final concentration of 20 mmol/L was added to the precipitate and centrifuged again. The precipitate was resuspended in the resuspension solution and stored at 4 °C.

### 2.4. Sample Pad and Gold Label Pad Processing

The sample and gold label pads were cut from glass fiber cotton and were immersed in the treatment solution for 1 min. The sample pad was placed in FZG-P vacuum drying box (Chengzao Inc., Shanghai, China) at 45 °C to dry for 1 h while the gold label pad was placed at 37 °C to dry for 4 h.

### 2.5. Test Strip Assembly

The gold-labeled antibody was sprayed on the gold-labeled pad by using the XYZ-3000 gold spray-point film meter (Bio-Dot Inc., Irvine, CA, USA) at a spraying volume of 5 μL/cm. The conjugated antigens AFB_1_–OVA and OTA–OVA were diluted to 0.5 mg/mL with 0.02 mol/L PBS solution. The spraying volume was 3 μL/cm at 9 mm and 13 mm from the top of the NC film (Shenzhen Tisenc Medical Devices Co. Ltd., Shenzhen, China), respectively, to form the detection lines (T_1_ and T_2_ lines). In addition, a goat anti-mouse IgG with a concentration of 0.5 mg/mL and a spray volume of 5 μL/cm was sprayed at 5 mm as a quality control line (C line). The gold label pad and NC film were dried at 37 °C for 2 h. Subsequently, the strip was assembled in the following order: the sample pad, the gold label pad, the NC film, and the absorbent pad (MIDWEST Inc., Beijing, China) were all located on the PVC bottom plate (Shenzhen Tisenc Medical Devices Co. Ltd., Shenzhen, China). Each adjacent pair of materials were overlapped by 2–3 mm and pressed tightly. Finally, this assembly was cut into 3 mm wide strips and placed in a plastic card case to make test strips.

### 2.6. Test Strip Performance Appraisal

#### 2.6.1. Test Strip Detection Limit Test

Mycotoxin standard solutions were added to negative methanol extracts of Chinese prickly ash, pepper, chili, cinnamon, and aniseed such that the final concentrations of AFB_1_ were 0, 3, 5, 6, 12, 25, and 50 μg/kg, and of OTA were 0, 5, 10, 15, 25, and 50 μg/kg. The extraction solution (30 μL) and sample buffer (150 μL) were mixed uniformly; 100 μL of this solution was added onto the test strip and incubated in GL-1700 dry thermostat (MIDWEST Inc., Beijing, China) at 37 °C for 10 min. Experiments were repeated five times per each concentration; the detection results were recorded by the iCheck-III card reader (Clover Technology Group Inc., Beijing, China).

#### 2.6.2. Test Strip Specific Test

The standard solutions of AFB_1_, AFB_2_, AFG_1_, AFG_2_, OTA, FB, DON, ZEN, and T_2_ were diluted with sample buffer. The concentrations of AFB_1_, OTA, FB, DON, ZEN, and T_2_ standard solution were 20 μg/kg, 20 μg/kg, 1 mg/kg, 1 mg/kg, 200 μg/kg, 100 μg/kg, respectively. Then, 100 μL of the solution was used for the reaction; each concentration was tested five times to determine the specificity of the test strip. For the verification of structural analogs, the concentrations of AFB_1_, AFB_2_, AFG_1_, and AFG_2_ standard solution were 0 μg/kg, 1 μg/kg, 5 μg/kg, 20 μg/kg, 50 μg/kg, 100 μg/kg, respectively. Then, 30 μL of the solution was used for the reaction, the detection results were recorded by the iCheck-III card reader and calculated IC50 and cross-reaction rate (Table S1).

#### 2.6.3. Test Strip Precision Test

AFB_1_ and OTA standard solutions were added to 5.0 g of Chinese prickly ash, pepper, chili, cinnamon, and aniseed negative samples so that the contents of the two mycotoxins in the samples were 5, 20, and 50 μg/kg, respectively. Each sample was tested three times and the test results were recorded.

#### 2.6.4. Test Strip Repeatability Test

Three batches of test strips were used to detect the extracts with final concentrations of 0 and 20 μg/kg of AFB_1_ and OTA. Each extract was tested six times to test the repeatability of the test strip.

#### 2.6.5. Test Strip Stability Test

The test strips and desiccant were stored at 4 °C, 28 °C, and 45 °C in sealed storage for six months. Every other month, a part of the test strips was used to detect the AFB_1_ and OTA mixed sample extracts with final concentrations of 0 and 20 μg/kg. Compared with the newly developed test strip, the color of the T-line became lighter or the color disappeared, indicating that the test strip had exceeded its shelf life.

#### 2.6.6. Test Strips to Detect Spice Samples

The 5.0 g sample was placed in a centrifuge tube containing 25 mL of anhydrous methanol and mixed on a shaker at a speed of 94× *g* for 10 min. Subsequently, the mixture was centrifuged at a speed of 4742× *g* for 3 min and filtered. From this, 30 μL of the filtrate was mixed with 150 μL of sample buffer; 100 μL of the mixed solution was applied onto the test strip. The reaction was carried out with a dry thermostat at 37 °C for 10 min. The test strip was placed in a card reader for detection.

### 2.7. HPLC Determination

Based on the research of Zhao et al.and with some modifications, the concentrations of AFB_1_ and OTA in spices were detected [24,25].

#### 2.7.1. Sample Extraction and Purification

Extracts were prepared by shaking 2.5 g of each sample in 25 mL of extraction solution for 30 min. After centrifugation at a speed of 4742× *g* and filtration, the filtrate was diluted 10-fold with the diluent solution. The pH of the solution was adjusted to 7, and the mixture was again filtered through a glass fiber filter paper. Table 1 shows the three solutions prepared for five types of spices.

For purification, 20 mL of the filtrate was added to a glass syringe and allowed to pass through the IAC column (Clover Technology Group Inc., Beijing, China) under air pressure at a flow rate of 1–2 drops per second. The column was washed with 10 mL eluent solution as well as 10 mL ultrapure water at a flow rate of 1–2 drops per second. Finally, the column was washed with 1.5 mL methanol and the eluted solution was collected.

#### 2.7.2. HPLC Conditions

For AF detection, the HPLC system was equipped with a post-column derivatization reactor (Shimadzu Corporation, Kyoto, Japan). The injection volume was 20 µL, and the flow rate was 0.8 mL/min.

Methanol–water (45:55, *v*/*v*) was used as the mobile phase for AF detection. The excitation and emission wavelengths for AF detection were 360 nm and 440 nm, respectively. Acetonitrile–water–acetic acid (99:99:2, *v*/*v*) was used as the mobile phase for OTA detection. The excitation and emission wavelengths for OTA detection were 333 nm and 477 nm, respectively.

#### 2.7.3. HPLC Linear Range and Detection Limit

A series of standard solutions of the two kinds of mycotoxins was prepared. A standard curve was plotted using the peak area of the target compound as the ordinate (y) and the mass concentration of each mycotoxin (ng/mL) as the abscissa (x). Based on the progressive dilution method, the signal-to-noise ratio was used as the detection limit and as the theoretical limit of quantification three and ten times, respectively [26].

#### 2.7.4. HPLC Recovery Test and Precision

Five kinds of negative spice samples were added with high, medium, and low concentration levels of mycotoxins for recovery tests. Each sample was tested in 6 copies in parallel to investigate the recovery and reproducibility of the method. In addition, 10 μg/kg standard solutions of two mycotoxins were prepared. The sample was injected once every three hours, and a total of 6 injections were made to measure the daytime precision of the instrument. For six consecutive days, samples were injected at the same time every day, and the intraday precision of the analytical instrument was analyzed.

## 3. Results and Discussion

### 3.1. Test Strip Performance

#### 3.1.1. Detection Limit and Specificity of Test Strips

**Figure 1:**
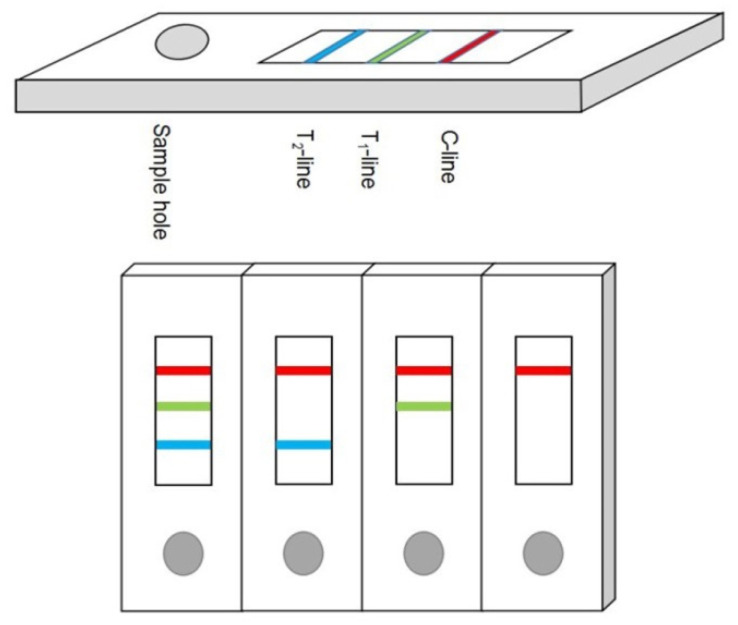
Schematic diagram of test strip structure and test results.

The external structure of the colloidal gold immuno-chromatographic test paper is shown in Figure 1, which consists of a sample hole, two T lines, and one C line. The C line is also known as the quality control line. The test results are valid only when the C line is colored. The T line is also called the detection limit. The degree of color development of the T-line is inversely proportional to the content of mycotoxins in the sample. In this study, the dilution factor of the sample buffer was increased for two reasons: the first is that the spice contained a complex matrix. Water-soluble impurities will dissolve in the inorganic phase of the extract. However, mycotoxins are extremely difficult to dissolve in water, so anhydrous methanol is used as the extraction solution. Since the tolerance of the antibody to methanol is approximately 20%, the filtrate was diluted six times. Another reason for this is that spices were rich in pigments and the T value would have been disturbed by the color in the extraction solution. Properly increasing the dilution factor can reduce the reading error caused by the pigment.

**Figure 2:**
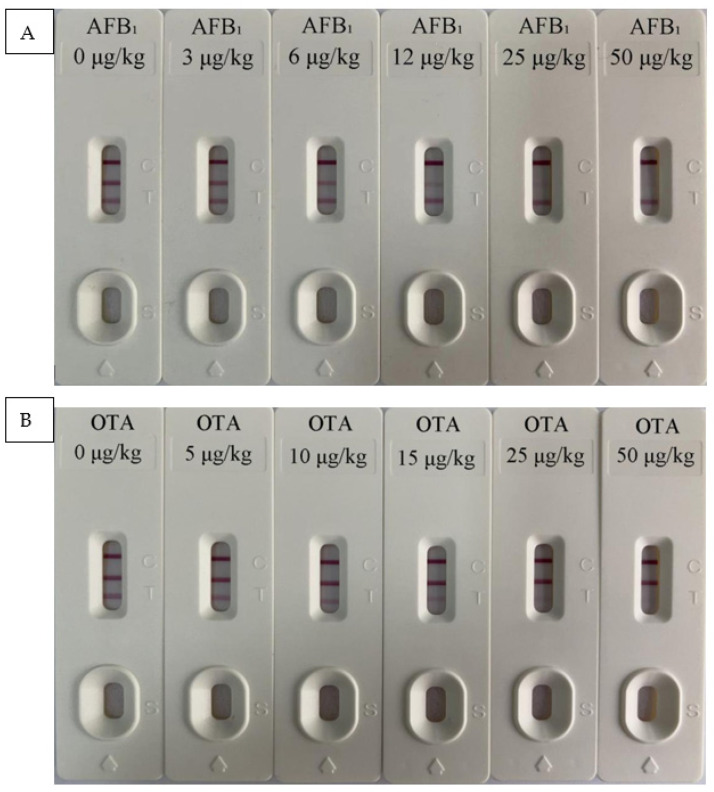
(**A**) aflatoxin B_1_ (AFB_1_) and (**B**) ochratoxin A (OTA) test strip quantitative detection limit determination.

**Figure 3:**
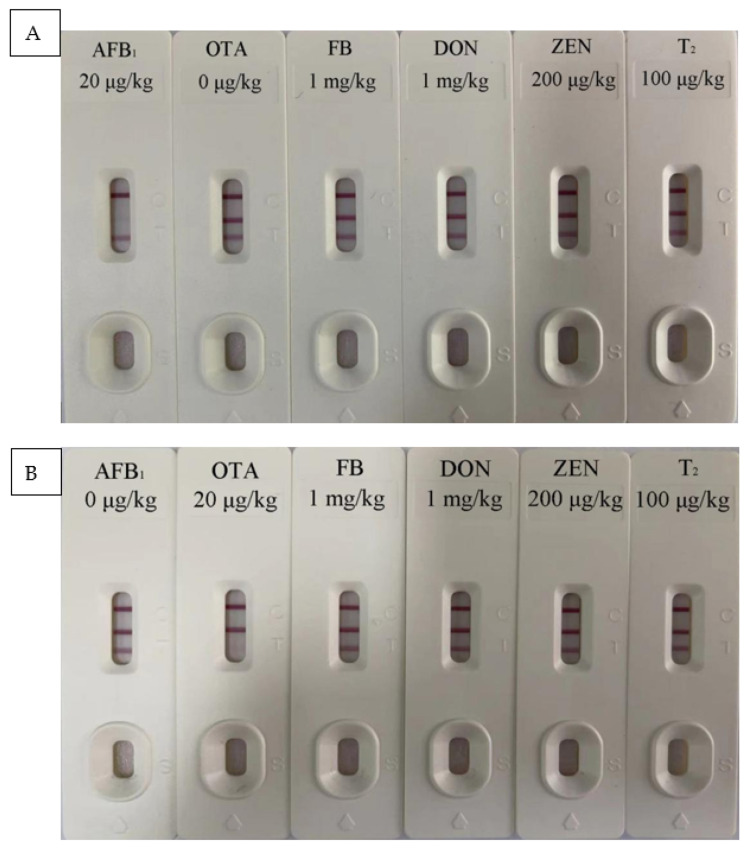
Test strip (**A**) T_1_ line and (**B**) T_2_ line specific test results.

Results of test strips in different spice bases are shown in Table 2 and Figure 2. When the mycotoxin concentration was 0 μg/kg, the two T lines showed a deep red color. When the concentration of AFB_1_ was 3 μg/kg, the color of T_1_ line became lighter which could be recorded by the iCheck-III card reader. When the OTA concentration was 5 μg/kg, the color of the T_2_ line could also be recorded by the card reader. As the concentration increases, the color further weakens. When the concentration reached 50 μg/kg, almost no red band was observed. Therefore, the detection limits of this test strip for AFB_1_ and OTA in Chinese prickly ash, pepper, chili, cinnamon, and aniseed were 3 and 5 μg/kg, respectively. The EU minimum limit standards for AFB_1_ and OTA in spices are 5 and 15 μg/kg, respectively. Therefore, the test strips developed in this study can meet the testing requirements in the EU and have a good market application prospect. Additionally, the test strip had a detection range of 0–50 μg/kg for the two mycotoxins. The results of the specificity test (Figure 3) showed that the T_1_ line only specifically binds to AFB_1_ and the T_2_ line only specifically binds to OTA. Moreover, the two T lines showed no cross-reactivity with other mycotoxins.

#### 3.1.2. Precision of Test Strips

**Figure 4:**
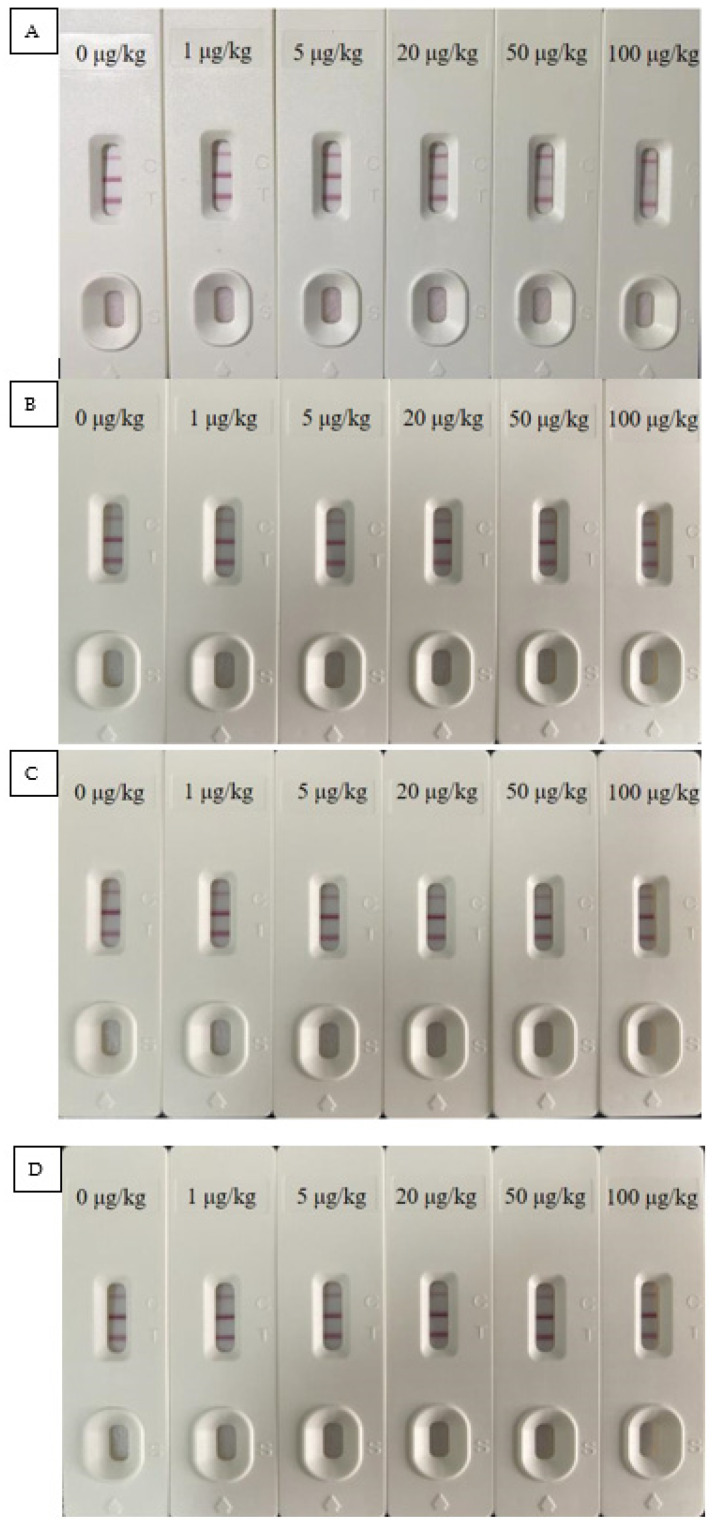
Verification of structural analogs test results, (**A**) AFB_1_, (**B**) AFB_2_, (**C**) AFG_1_, and (**D**) AFG_2_.

The precision of the test strip was reflected by the results of the added recovery test. It can be seen from Table 3 that the recovery rate of AFB_1_ was 81.2–113.7% with a relative standard deviation (RSD) of less than 9.2%. The recovery rate of OTA was in the range of 82.2–118.6% with an RSD of less than 8.7%. In accordance with regulatory limits, a recovery rate between 80.0–120.0% with an RSD of less than 10% is considered acceptable. From Table S1 and Figure 4, it can be seen that the cross-reaction rates of the T_1_ line to AFB_1_, AFB_2_, AFG_1_, and AFG_2_ were 100.0%, 7.7%, 4.2%, and less than 1.0%, respectively, and the T_2_ line had no cross-reactivity with these three structural analogs. This also means that if there are AFB_1_ structural analogues (AFB_2_, AFG_1_, and AFG_2_) in the actual spice sample without AFB_1_, the test strip may show a positive result, but after confirmation by the HPLC method, it can be ensured that there will be no false negative results in the detection of AFB_1_ in spices, thereby avoiding the possibility of missing the spice sample contaminated by AFB_1_.

#### 3.1.3. Repeatability and Stability of Test Strips

**Figure 5:**
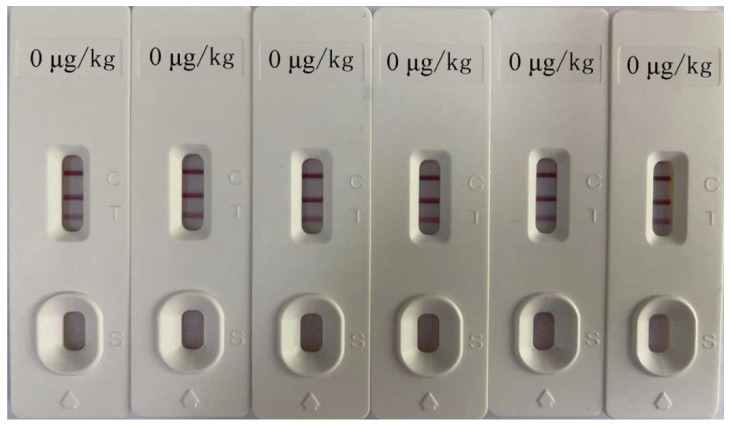
Test strip repeatability test results.

Figure 5 shows the test strip test results when the AFB_1_ and OTA concentrations were 0 μg/kg. It can be seen that the test strip had good repeatability. Moreover, the calculation results showed that the maximum coefficient of variation (CV) of the test strip was 4.28 (between batches) and 5.72% (within batches), respectively. No false positives or false negatives were observed, indicating that the test strip was highly repeatable. After the test strip was placed at 4 °C and 28 °C for 6 months, a clear red band was still visible as seen in the test results. However, when placed at 45 °C for 2 months, the color of the bands was significantly weakened. The detection results showed 5% false negatives upon storage in these conditions. According to the calculation result of the Arrhenius formula, the storage of test strips at 45 °C for 37.5 days is equivalent to storage at 25 °C for 12 months [27]. Therefore, the shelf life of the developed test strip is 6 months when seal-dried and stored at 4–28 °C.

### 3.2. Immuno-Chromatographic Standard Curve

Three negative samples were randomly selected for each kind of spice. AFB_1_ and OTA mixed with standard solutions were added to obtain spiked samples with final concentrations of 0, 5, 10, 20, and 50 μg/kg. After the reaction was completed, the values of the T lines were recorded. Each concentration was tested five times in parallel and the average value was used to draw a standard curve. This was used as the built-in curve of the card reader. The standard curve equation and correlation coefficient (*R*^2^) are shown in Table 4. It can be seen from the table that *R*^2^ was greater than 0.96, which means that the newly developed detection method is reliable.

### 3.3. HPLC Verification

Table 5 shows the linear regression equation of the standard curve of HPLC. The correlation coefficients (*R*^2^) were all greater than 0.9998, indicating that the standard curve had a good linearity. After calculation, the detection limit of mycotoxins was between 0.14–0.21 µg/kg and the limit of quantification was between 0.47–0.70 µg/kg. The relative standard deviation of intra-day and inter-day precision tests were within the range of 0.3–1.8% (Table 6). This indicates that the instrument had good stability and high reliability. The recoveries of the two mycotoxins at the low, medium, and high levels of the standard addition were between 82.6% and 117.0%. The relative standard deviation was 2.5–8.7% (Table 7). Zhao et al. developed a systematic analysis method to detect AFs and OTA in fragrances [24,25]. The average recovery rate of the samples was between 78.0–96.6%. In this study, we replaced the extraction solution used for the detection of AFs from ethanol to acetonitrile; the methanol content of the extraction solution was increased for OTA detection. Under these conditions, the average recovery rate was 82.6–117.0%. This may be because AFs are highly polar compounds; thus, the solubility in acetonitrile is higher than that in ethanol. Therefore, after the methanol ratio increased, the recovery rate was also increased.

A total of 150 spice samples (including Chinese prickly ash, pepper, chili, cinnamon, and aniseed, 30 samples of each) were tested by GICA and HPLC. The test results of positive samples are shown in Table 8. It can be seen from the table that the results of GICA and HPLC show the same trend, which suggests that the developed test strip has high accuracy and can be used for sample detection. While increasing the dilution factor of the extract can reduce the influence of matrix effects on the test results, there was still a 2% false positive rate. Follow-up research can help develop better filtration, adsorption, and other methods to further improve the accuracy of test strips. Combined data analysis showed that, overall, 17 samples were contaminated by mycotoxins. Only one chili sample was contaminated by both AFB_1_ and OTA. The AFB_1_ in one pepper sample and the OTA content in two chili samples exceeded the maximum permissible limit set by the European Union, at 14.1, 84.3, and 26.1 μg/kg, respectively. From the analysis of sample types, the contamination rate of pepper was the highest (30%). A study by Bircan showed that chili powder is more susceptible to AFB_1_ contamination than other spices [28]. Another study used HPLC-FLD to test 70 pepper samples from Hungary, and the results were consistent with the results of this study [29]. None of the cinnamon samples in this study were contaminated by mycotoxins. AFB_1_ was also not detected in Irish cinnamon samples [30].

## 4. Conclusions

AFB_1_ and OTA colloidal gold rapid synchronous detection test strips were developed and an effective sample preparation method was established, the results for which could be obtained within ten minutes without any equipment. The average recovery rate of the two mycotoxins was 81.2–118.6%. Although there were false positives in the detection process, the false negative rate was zero. Moreover, validation by HPLC analysis confirmed that the test strip immunoassay was reliable. Compared with HPLC, the GICA detection cost is low and takes one-third of the time. Therefore, it is more suitable for large-scale commercial testing. Additionally, this study analyzed five kinds of spice samples; follow-up research should expand the types of spices that can be tested effectively to improve the reliability and practicability of this detection method.

After testing, a total of 17 spice samples were found to be contaminated with mycotoxins, which exceeded 10% of the total number of samples. Among them, the maximum limit of mycotoxins stipulated by the European Union was exceeded in three samples. Given the dangers of mycotoxins, this poses a threat to food safety. Mycotoxins can be produced during various stages, such as crop planting, processing, storage, and transportation. The amounts of mycotoxins vary with the types of spices, areas they are produced in, and sales areas. To reduce the possible health hazards caused by such contaminated spices in humans, relevant regulatory agencies must strengthen supervision. At the same time, it is important to actively improve the environmental conditions before the spices are sold and increase the number of inspections to prevent contaminated spices from entering the market.

## Figures and Tables

**Table 1:** Solution used for HPLC pretreatment.

Spices	AFB_1_ Pretreatment	OTA Pretreatment		
	Chinese Prickly Ash, Pepper, Chili, Cinnamon, Aniseed	Chinese Prickly Ash, Pepper, Chili	Cinnamon	Aniseed
Extraction solution	Acetonitrile–water (80:20, *v/v*)	Methanol–1% NaHCO_3_ (70:30, *v/v*)	Methanol	Methanol–1% NaHCO_3_ (70:30, *v/v*)
Dilute solution	Tween–PBS (5:95, *v/v*)	Tween–PBS (1:99, *v/v*)		
Rinse solution	Water	PBS		

Note: AFB_1_, aflatoxin B_1_; OTA, ochratoxin A.

**Table 2:** Detection limit of test strip.

Spices	Mass Concentration of AFB_1_ Standard Solution (μg/kg)						Mass Concentration of OTA Standard Solution (μg/kg)					
	0	3	6	12	25	50	0	5	10	15	25	25
Chinese prickly ash	−	+	+	+	+	+	−	+	+	+	+	+
Pepper	−	+	+	+	+	+	−	+	+	+	+	+
Chili	−	+	+	+	+	+	−	+	+	+	+	+
Cinnamon	−	+	+	+	+	+	−	+	+	+	+	+
Aniseed	−	+	+	+	+	+	−	+	+	+	+	+

Note: “−” means negative, “+” means positive; AFB_1_, aflatoxin B_1_; OTA, ochratoxin A.

**Table 3:** Precision of test strips.

Mycotoxins	Add Level (µg/kg)	Chinese Prickly Ash		Pepper		Chili		Cinnamon		Aniseed	
		Recovery Rate (%)	RSD (%)	Recovery Rate (%)	RSD (%)	Recovery Rate (%)	RSD (%)	Recovery Rate (%)	RSD (%)	Recovery Rate (%)	RSD (%)
AFB_1_	5.0	83.2 ± 2.8	2.6	81.2 ± 6.6	6.1	105.3 ± 2.9	3.6	100.4 ± 2.8	3.3	111.0 ± 5.6	5.9
	20.0	102.8 ± 3.9	4.3	110.0 ± 2.7	3.5	110.2 ± 9.3	9.2	103.4 ± 5.2	5.9	83.6 ± 6.8	6.2
	50.0	93.2 ± 6.3	7.5	99.1 ± 2.0	2.8	113.7 ± 3.7	4.1	92.9 ± 6.9	6.3	110.4 ± 2.4	2.6
OTA	5.0	101.2 ± 4.6	3.8	95.8 ± 4.8	4.2	109.4 ± 5.8	6.5	82.2 ± 7.4	8.7	118.6 ± 2.7	3.5
	20.0	113.0 ± 5.1	5.3	109.3 ± 2.6	1.1	100.7 ± 2.9	3.0	103.0 ± 2.1	3.1	86.8 ± 4.6	5.1
	50.0	107.6 ± 2.9	4.6	92.1 ± 4.5	4.8	99.3 ± 6.4	7.5	91.7 ± 3.2	4.8	91.4 ± 3.4	4.9

Note: RSD, relative standard deviation; AFB_1_, aflatoxin B_1_; OTA, ochratoxin A.

**Table 4:** Standard curve of different spice bases.

Spices	AFB_1_		OTA	
	Regression Equation	*R* ^2^	Regression Equation	*R* ^2^
Chinese prickly ash	y = 0.5635x − 0.3801	0.9982	y = 0.6090x − 0.3714	0.9912
Pepper	y = 0.5569x − 0.3638	0.9627	y = 0.5797x − 0.4222	0.9621
Chili	y = 0.6120x − 0.3332	0.9695	y = 0.6574x − 0.4537	0.9887
Cinnamon	y = 0.4551x − 0.3148	0.9729	y = 0.6127x − 0.4276	0.9638
Aniseed	y = 0.6169x − 0.4767	0.9657	y = 0.6445x − 0.4689	0.9763

Note: AFB_1_, aflatoxin B_1_; OTA, ochratoxin A.

**Table 5:** HPLC regression equation, correlation coefficient, and linear range (µg/kg).

Mycotoxins	Regression Equation	*R* ^2^	Linearity Range (µg/kg)
AFB_1_	y = 75,941x + 40,497	0.9998	1.0–200.0
OTA	y = 20,810x − 2861.3	0.9998	1.0–200.0

Note: AFB_1_, aflatoxin B_1_; OTA, ochratoxin A.

**Table 6:** HPLC instrument precision.

Mycotoxins	Intraday Precision		Daytime Precision	
	Recovery Rate (%)	RSD (%)	Recovery Rate (%)	RSD (%)
AFB_1_	105.7 ± 1.7	1.6	98.2 ± 1.4	1.4
OTA	100.5 ± 1.5	1.5	108.1 ± 1.9	1.8

Note: AFB_1_, aflatoxin B_1_; OTA, ochratoxin A; RSD, relative standard deviation.

**Table 7:** HPLC addition recovery rate and relative standard deviation.

Mycotoxins	Add Level (µg/kg)	Chinese Prickly Ash		Pepper		Chili		Cinnamon		Aniseed	
		Recovery Rate (%)	RSD (%)	Recovery Rate (%)	RSD (%)	Recovery Rate (%)	RSD (%)	Recovery Rate (%)	RSD (%)	Recovery Rate (%)	RSD (%)
AFB_1_	2.0	117.0 ± 6.8	5.8	111.4 ± 9.3	8.3	96.7 ± 8.4	8.7	116.3 ± 9.2	7.9	102.4 ± 4.8	4.7
	4.0	112.8 ± 5.1	4.5	85.7 ± 4.0	4.9	84.2 ± 5.7	6.8	105.3 ± 6.7	6.4	107.6 ± 5.3	4.9
	20.0	108.2 ± 3.2	3.0	82.6 ± 3.1	3.7	113.9 ± 3.2	2.8	84.5 ± 3.7	4.4	98.4 ± 7.2	7.3
OTA	2.0	102.1 ± 5.3	5.2	93.5 ± 4.7	5.0	94.8 ± 3.6	3.8	102.6 ± 4.2	4.1	96.3 ± 5.8	6.0
	4.0	97.7 ± 2.4	2.5	104.3 ± 2.9	2.8	99.1 ± 5.7	5.8	92.7 ± 6.3	6.8	104.4 ± 6.1	5.8
	20.0	105.8 ± 4.9	4.6	109.2 ± 6.1	5.6	112.7 ± 4.3	3.8	102.3 ± 5.8	5.7	93.7 ± 3.7	3.9

Note: AFB_1_, aflatoxin B_1_; OTA, ochratoxin A; RSD, relative standard deviation.

**Table 8:** Comparison of GICA and HPLC detection methods.

Mycotoxins	Serial Number	GICA (μg/kg)	HPLC (μg/kg)	Mycotoxins	Serial Number	GICA (μg/kg)	HPLC (μg/kg)
AFB_1_	19-1	3.2 ± 0.2	3.1 ± 0.3	OTA	19-3	3.8 ± 0.6	3.9 ± 0.4
	22-1	2.9 ± 0.5	2.8 ± 0.2		20-3	5.7 ± 0.3	5.4 ± 0.7
	25-2	14.4 ± 0.4	14.1 ± 0.5		22-3	3.1 ± 0.5	3.5 ± 0.2
	2-3	5.0 ± 0.3	4.8 ± 0.7		26-3	28.6 ± 2.7	26.1 ± 1.3
	28-3	4.1 ± 0.2	4.3 ± 0.4		28-3	3.8 ± 0.4	4.2 ± 0.7
OTA	23-1	3.6 ± 0.5	3.2 ± 0.6		30-3	6.5 ± 0.2	6.6 ± 0.6
	8-2	4.4 ± 0.3	4.7 ± 0.8		2-5	7.4 ± 1.0	7.2 ± 0.9
	4-3	81.9 ± 3.6	84.3 ± 1.5		4-5	5.7 ± 0.3	6.1 ± 0.1
	12-3	3.5 ± 0.4	3.4 ± 0.2		16-5	12.9 ± 0.8	13.5 ± 0.8

Note: AFB_1_, aflatoxin B_1_; OTA, ochratoxin A; GICA, colloidal gold immuno-chromatographic assays.

## Data Availability

Not applicable.

## Supplementary Materials

The following are available online at https://www.mdpi.com/article/10.3390/foods10112738/s1, Table S1: Cross-reaction experiment data of test strips.

## Abbreviations

(AFs)	aflatoxins
(AFB_1_)	aflatoxin B_1_
(OTA)	ochratoxin A
(AFB_1_–OVA)	AFB_1_–ovalbumin
(OTA–OVA)	OTA–ovalbumin
(RSD)	relative standard deviation
(ELISA)	enzyme-linked immunosorbent assay
(HPLC)	high performance liquid chromatography
(HPLC–MS)	HPLC–mass spectrometry
(GICA)	colloidal gold immuno-chromatography assay
(ICA)	immuno-chromatographic analysis
(ZEN)	zearalenone
(T_2_)	trichothecenes
(DON)	deoxynivalenol
(FB) standard solutions	fumonisins
IgG	goat anti-mouse
BSA	bovine serum albumin
