# Supplementary material for: Simultaneous Rapid Detection of Aflatoxin B1 and Ochratoxin A in Spices Using Lateral Flow Immuno-Chromatographic Assay
Source: Foods. 2021 Nov 9;10(11):2738. doi: 10.3390/foods10112738 (PMC8623159; doi:10.3390/foods10112738)
Supplement: Supplementary file 1 [file foods-10-02738-s001.zip › foods-1461107-supplementary.pdf]

foods

MDPI

Supplementary material

Simultaneous Rapid Detection of Aflatoxin B1 and Ochratoxin A in Spices Using Lateral Flow Immuno-Chromatographic Assay

Xue Zhao [1,†], Xindi Jin [1,†], Zhang Lin [1], Qi Guo [1], Bin Liu [1,2], Yahong Yuan [1,2], Tianli Yue [1,2] and Xubo Zhao [1,2*]

[1] College of Food Science and Engineering, Northwest A&F University, No. 22 Xinong Road, Yangling, Shaanxi 712100, China; 15735172570@163.com (X.Z.); xd.jin@nwafu.edu.cn (X.J.); linzhang@nwafu.edu.cn (Z.L.); guoqiqi@nwafu.edu.cn (Q.G.); liubin7723@163.com (B.L.); yyh324@tom.com (Y.Y.); yuetl@nwafu.edu.cn (T.Y.)
[2] Laboratory of Quality & Safety Risk Assessment for Agro-Products (Yangling), Ministry of Agriculture and Rural Affairs, Yangling, Shaanxi 712100, China
* Correspondence: zhxubo@nwafu.edu.cn
† These authors contributed equally to this work

Citation: Zhao, X.; Jin, X.; Lin, Z.; Guo, Q.; Liu, B.; Yuan, Y.; Yue, T.; Zhao, X. Simultaneous Rapid Detection of Aflatoxin B1 and Ochratoxin A in Spices Using Lateral Flow Immuno-Chromatographic Assay. *Foods* 2021, 10, 2738. https://doi.org/10.3390/foods10112738

Academic Editor: Cristina A. Fente

Received: 28 October 2021 Accepted: 4 November 2021 Published: date

Publisher's Note: MDPI stays neutral with regard to jurisdictional claims in published maps and institutional affiliations.

Copyright: © 2021 by the authors. Submitted for possible open access publication under the terms and conditions of the Creative Commons Attribution (CC BY) license (http://creativecommons.org/licenses/by/4.0/).

Table S1. Cross-reaction experiment data of test strips.

Structural analogs	Photometric value	Add level (μg/L)						IC50 (μg/L)	Cross reaction rate (%)
		0.0	1.0	5.0	20.0	50.0	100.0		
G_2	Control	12.11	12.16	15.10	12.12	11.15	15.17	-	-
	AFB_1	165.24	164.22	170.14	170.10	164.12	172.29	> 1000.0	<1
	OTA	136.21	136.18	136.13	135.29	130.23	128.16	-	-
G_1	Control	12.17	12.27	12.21	13.15	23.11	73.19	-	-
	AFB_1	164.06	169.14	167.24	170.12	166.27	130.15	278.6	4.20
	OTA	130.28	129.13	131.12	135.19	132.13	132.11	-	-
B_2	Control	14.13	12.19	12.17	13.16	64.19	128.25	-	-
	AFB_1	169.18	169.20	172.11	170.13	130.26	100.17	151.1	7.7
	OTA	132.25	131.15	135.19	134.26	135.11	131.13	-	-
B_1	Control	12.10	55.12	108.16	165.12	185.16	193.20	-	-
	AFB_1	173.13	145.11	110.20	70.16	42.09	25.12	11.6	100.0
	OTA	132.23	135.26	130.17	132.27	131.12	136.19	-	-
